# Supplementary material for: RNA Profile of Cell Bodies and Exosomes Released by Tumorigenic and Non-Tumorigenic Thyroid Cells
Source: Int J Mol Sci. 2024 Jan 24;25(3):1407. doi: 10.3390/ijms25031407 (PMC10855121; doi:10.3390/ijms25031407)
Supplement: Supplementary file 1 [file ijms-25-01407-s001.zip › Supplemtary Tables S1 and S2.pdf]

**Table S1. Top 20 de-regulated molecular pathway in TPC1.**

| UP-REGULATED | | | DOWN-REGULATED | | |
|---|---|---|---|---|---|
| **GO term** | **Description** | **FDR q-value** | **GO term** | **Description** | **FDR q-value** |
| GO:0050911 | detection of chemical stimulus involved in sensory perception of smell | 4.03E-306 | GO:0044237 | cellular metabolic process | 1.07E-118 |
| GO:0050907 | detection of chemical stimulus involved in sensory perception | 1.49E-304 | GO:0008152 | metabolic process | 5.94E-102 |
| GO:0009593 | detection of chemical stimulus | 9.38E-279 | GO:0006807 | nitrogen compound metabolic process | 4.10E-97 |
| GO:0050906 | detection of stimulus involved in sensory perception | 8.31E-269 | GO:0071704 | organic substance metabolic process | 8.65E-95 |
| GO:0051606 | detection of stimulus | 2.52E-208 | GO:0044238 | primary metabolic process | 6.60E-94 |
| GO:0007186 | G protein-coupled receptor signaling pathway | 1.54E-166 | GO:0043170 | macromolecule metabolic process | 6.42E-91 |
| GO:0031424 | keratinization | 7.53E-43 | GO:0044260 | cellular macromolecule metabolic process | 1.66E-88 |
| GO:0007608 | sensory perception of smell | 2.28E-42 | GO:0034641 | cellular nitrogen compound metabolic process | 1.71E-60 |
| GO:0007606 | sensory perception of chemical stimulus | 1.97E-39 | GO:0006139 | nucleobase-containing compound metabolic process | 7.59E-59 |
| GO:0007165 | signal transduction | 1.40E-28 | GO:0044267 | cellular protein metabolic process | 5.68E-57 |
| GO:0050896 | response to stimulus | 2.20E-12 | GO:0046483 | heterocycle metabolic process | 6.89E-57 |
| GO:0033141 | positive regulation of peptidyl-serine phosphorylation of STAT protein | 1.41E-09 | GO:0071840 | cellular component organization or biogenesis | 1.00E-54 |
| GO:0033139 | regulation of peptidyl-serine phosphorylation of STAT protein | 4.77E-08 | GO:0090304 | nucleic acid metabolic process | 1.07E-54 |
| GO:0001580 | detection of chemical stimulus involved in sensory perception of bitter taste | 6.66E-08 | GO:0043412 | macromolecule modification | 1.21E-54 |
| GO:0006342 | chromatin silencing | 6.92E-08 | GO:0006725 | cellular aromatic compound metabolic process | 2.93E-54 |

| | | | | | |
|---|---|---|---|---|---|
| GO:0002323 | natural killer cell activation involved in immune response | 1.05E-07 | GO:0051641 | cellular localization | 3.48E-54 |
| GO:0006334 | nucleosome assembly | 1.55E-07 | GO:0016043 | cellular component organization | 4.33E-52 |
| GO:0050912 | detection of chemical stimulus involved in sensory perception of taste | 2.39E-07 | GO:1901564 | organonitrogen compound metabolic process | 3.03E-50 |
| GO:0007600 | sensory perception | 3.15E-07 | GO:1901360 | organic cyclic compound metabolic process | 2.94E-50 |
| GO:0045814 | negative regulation of gene expression, epigenetic | 3.01E-06 | GO:0006464 | cellular protein modification process | 8.37E-50 |

## Table S2. Top 20 de-regulated molecular pathway in NThy-ori 3.1.

| **UP-REGULATED** | | | **DOWN-REGULATED** | | |
|---|---|---|---|---|---|
| **GO term** | **Description** | **FDR q-value** | **GO term** | **Description** | **FDR q-value** |
| GO:0050911 | detection of chemical stimulus involved in sensory perception of smell | 2.52E-285 | GO:0071840 | cellular component organization or biogenesis | 2.41E-36 |
| GO:0050907 | detection of chemical stimulus involved in sensory perception | 2.41E-280 | GO:0016043 | cellular component organization | 1.21E-36 |
| GO:0009593 | detection of chemical stimulus | 8.24E-255 | GO:0044237 | cellular metabolic process | 1.41E-30 |
| GO:0050906 | detection of stimulus involved in sensory perception | 3.80E-243 | GO:0006807 | nitrogen compound metabolic process | 3.24E-30 |
| GO:0051606 | detection of stimulus | 1.27E-182 | GO:0044260 | cellular macromolecule metabolic process | 2.87E-30 |
| GO:0007186 | G protein-coupled receptor signaling pathway | 1.43E-153 | GO:0043170 | macromolecule metabolic process | 2.26E-28 |
| GO:0031424 | keratinization | 1.37E-42 | GO:0044238 | primary metabolic process | 2.77E-28 |
| GO:0007608 | sensory perception of smell | 8.32E-41 | GO:0006996 | organelle organization | 3.09E-28 |
| GO:0007606 | sensory perception of chemical stimulus | 4.96E-38 | GO:0071704 | organic substance metabolic process | 6.59E-27 |
| GO:0007165 | signal transduction | 1.05E-28 | GO:0008152 | metabolic process | 3.24E-26 |

| | | | | | |
|---|---|---|---|---|---|
| GO:0050896 | response to stimulus | 7.98E-10 | GO:0051641 | cellular localization | 1.81E-22 |
| GO:0007600 | sensory perception | 2.00E-07 | GO:0022402 | cell cycle process | 2.77E-20 |
| GO:0033141 | positive regulation of peptidyl-serine phosphorylation of STAT protein | 4.70E-06 | GO:1903047 | mitotic cell cycle process | 2.84E-20 |
| GO:0050912 | detection of chemical stimulus involved in sensory perception of taste | 2.81E-05 | GO:0033043 | regulation of organelle organization | 4.25E-20 |
| GO:0001580 | detection of chemical stimulus involved in sensory perception of bitter taste | 5.02E-05 | GO:0006464 | cellular protein modification process | 3.00E-19 |
| GO:0033139 | regulation of peptidyl-serine phosphorylation of STAT protein | 5.58E-05 | GO:0036211 | protein modification process | 2.81E-19 |
| GO:0002323 | natural killer cell activation involved in immune response | 1.11E-04 | GO:0051128 | regulation of cellular component organization | 5.91E-19 |
| GO:0050877 | nervous system process | 4.40E-04 | GO:0043412 | macromolecule modification | 8.69E-19 |
| GO:0098664 | G protein-coupled serotonin receptor signaling pathway | 9.03E-04 | GO:0090304 | nucleic acid metabolic process | 4.07E-17 |
| GO:0007210 | serotonin receptor signaling pathway | 1.57E-03 | GO:0006259 | DNA metabolic process | 2.99E-16 |
