# Supplementary figures and images for: RNA Profile of Cell Bodies and Exosomes Released by Tumorigenic and Non-Tumorigenic Thyroid Cells
Source: Int J Mol Sci. 2024 Jan 24;25(3):1407. doi: 10.3390/ijms25031407 (PMC10855121; doi:10.3390/ijms25031407)

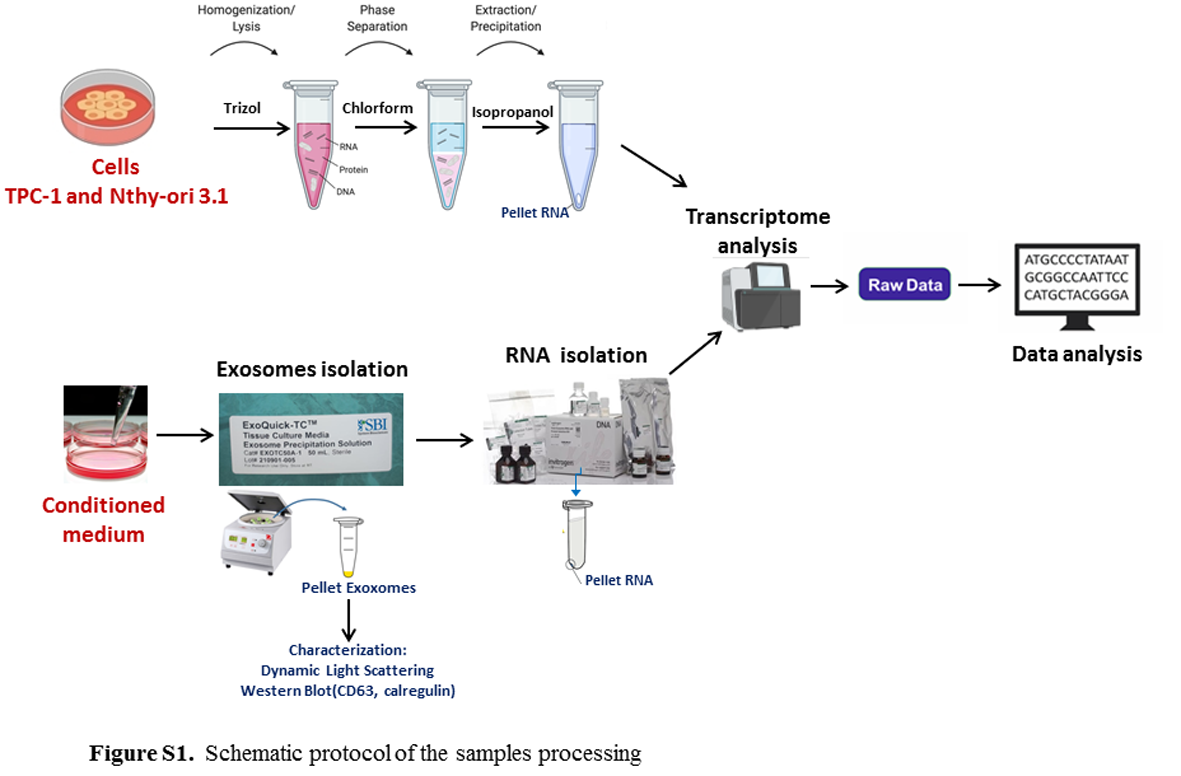

Supplement: Supplementary file 1 [file ijms-25-01407-s001.zip › Supplementary Figure S1.tif]

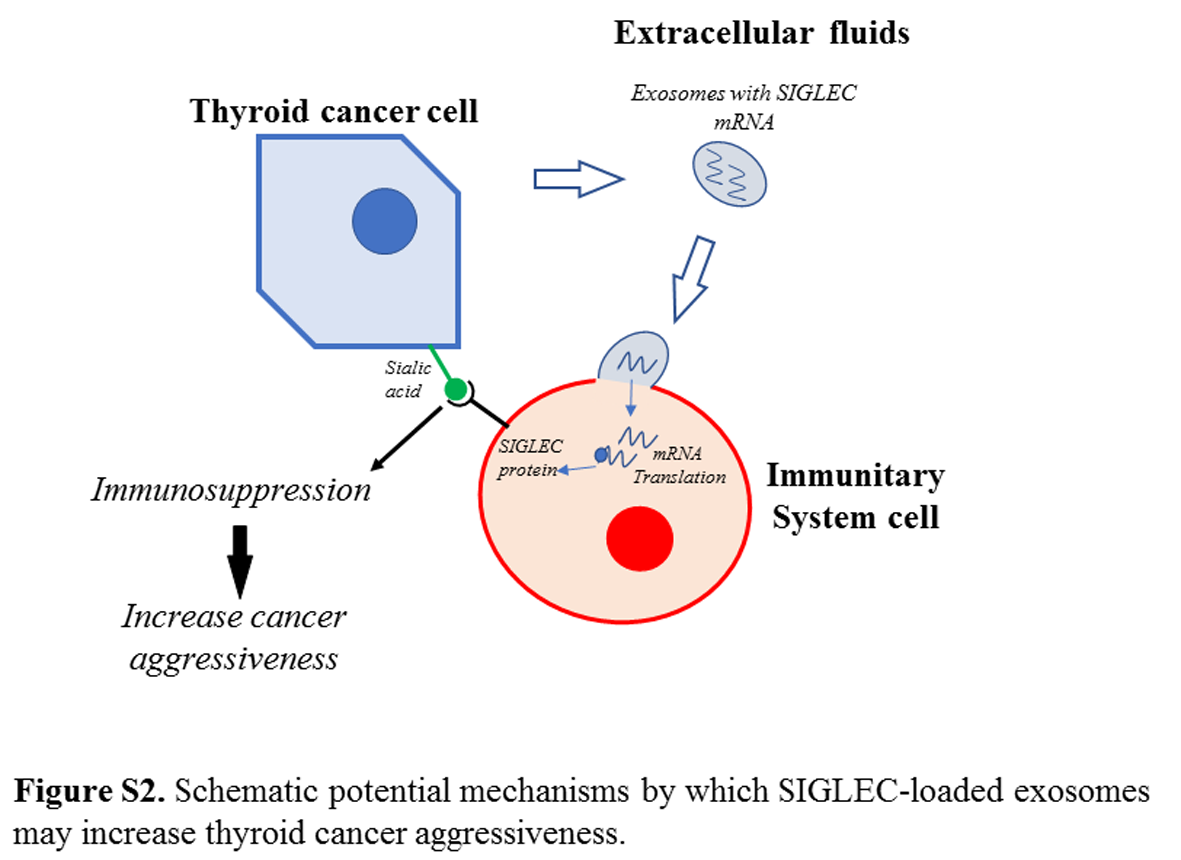

Supplement: Supplementary file 1 [file ijms-25-01407-s001.zip › Supplementary Figure S2.tif]
